# Reduced mitochondrial DNA content correlate with poor clinical outcomes in cryotransfers with day 6 single euploid embryos

**DOI:** 10.3389/fendo.2022.1066530

**Published:** 2023-01-04

**Authors:** Tzu-Hsuan Chuang, Chih-Yen Chen, Chin-Sheng Kuan, Hsing-Hua Lai, Chia-Lin Hsieh, Meng-Ju Lee, Yi-Ting Liang, Yu-Jen Chang, Chien-Yu Chen, Shee-Uan Chen

**Affiliations:** ^1^ Stork Fertility Center, Stork Ladies Clinic, Hsinchu, Taiwan; ^2^ Graduate Institute of Clinical Medicine, National Taiwan University and College of Medicine, Taipei, Taiwan; ^3^ Bioresource Collection and Research Center, Food Industry Research and Development Institute, Hsinchu, Taiwan; ^4^ Department of Biomechatronics Engineering, National Taiwan University, Taipei, Taiwan; ^5^ Department of Obstetrics and Gynecology, National Taiwan University Hospital and College of Medicine, Taipei, Taiwan

**Keywords:** mitochondria, blastocyst formation day, preimplantation genetic testing for aneuploidy(PGT-A), next-generation sequencing, single embryo transfer (SET)

## Abstract

**Objective:**

To investigate whether the mitochondrial DNA (mtDNA) content of a single biopsy at trophoblast correlates with the developmental potential and reproductive outcomes of blastocyst.

**Methods:**

A retrospective analysis applied the dataset of 1,675 embryos with preimplantation genetic testing for aneuploidy (PGT-A) from 1,305 individuals, and 1,383 embryos involved cryotransfers of single euploid embryo between January 2015 and December 2019. The studied cohort was divided for algorithm establishment on the NGS platform (n=40), correlation of biological features (n=1,635), and correlation of reproductive outcomes (n=1,340). Of the algorithm derived from the NGS platform, the reliability and repeatability were validated *via* qPCR assay and inter-run controls, respectively. Of the correlation across biological features, stratification analyses were applied to evaluate the effect from a single contributor. Eventually, the correlation between the mtDNA ratios and reproductive outcomes was adjusted according to the significant effector(s).

**Results:**

The mtDNA ratios showed statistically different between embryos with different days of blastocyst formation ([Day 5]: 1.06 vs. [Day 6]: 0.66, p=0.021), and between embryos with different expansion stages ([Expansion 5]: 1.05 vs. [Expansion 6]: 0.49, p=0.012). None or weakly correlated with the maternal age, morphology, ploidy, and gender. Analyzed by the different days of blastocyst formation with fixed expansion score as 5 in the euploid single embryo transfers (eSET), the day 6 eSET showed significantly lower reduced mtDNA ratio (n=139) in failure groups of fetal heartbeat (p=0.004), ongoing pregnancy (p=0.007), and live birth (p=0.01); however, no correlation between mtDNA ratios and pregnancy outcomes was observed in the day 5 eSET (n=1,201).

**Conclusions:**

The study first demonstrated that mtDNA ratio was dependent on the days of blastocyst formation while expansion stage was fixed. Lower mtDNA ratios were observed in the day 6 eSET with adverse outcomes. The present stratification analyses reveal that the timeline of embryo is an important covariate to the mtDNA content.

## Introduction

Chromosome aneuploidy is important to growth arrest of embryos, implantation failure, early miscarriage, and late abortion during *in-vitro* fertilization (IVF) program (1). Since the technique of preimplantation genetic testing for aneuploidy (PGT-A) was applied in the IVF for embryo selection, the overall implantation rate per transfer has been significantly improved (2) (3). However, failure of implantation and miscarriage still occurs in a percentage of both morphologically and chromosomally normal embryos, implying that other substantial factors could determine the embryo potential (4) (5). To date, the most prevalent procedure of PGT-A is to test multi-cellular biopsies of blastocyst trophectoderm *via* the methodologies of next-generation sequencing (NGS). Additional information can be immediately obtained from these trophoblasts *via* PGT-A/NGS. Accordingly, energy supply is an issue for embryo implantation, and the correlation between mitochondria and reproductive outcome deserves to be investigated (6, 7).

Mitochondrial DNA (mtDNA) is a double-stranded circular molecule, consisting of 16,569 base pairs (8). A total of 37 genes are contained, and make mitochondria the multifunctional powerhouse, which produce adenosine triphosphate (ATP) *via* the chain of oxidative phosphorylation (9–11). Moreover, mitochondria also involve cellular regulations, such as apoptosis, calcium homeostasis, and steroidogenesis in the ovaries (12, 13). Thus the amount of mitochondria in the human germline displays a very unique and dynamic pattern. The mitochondrial copy number in the oogonia is around 10-100, while it grows to approximately 150,000-700,000 in the metaphase II (MII) oocyte at the end of folliculogenesis. High amount of mitochondria makes the mature oocyte be ready for following fertilization and development, which require sufficient energy supply. Then the proliferation of mitochondria is ceased during the post-fertilization stage, and the existing mtDNAs are subsequently distributed to the divided blastomeres, from approximately 80,000 copies per blastomere in the four-cell stage to 1,000 copies per cell in the blastocysts (14).

Since the structure of mtDNA lacks an effective repairing system as nuclear DNA, and the polymerase γ (polymerase of mtDNA) has comparatively poorer ability of proofreading, the mutation rate of mtDNA is around tenfold higher than that of nuclear DNA. Moreover, mitochondria are the major site of generating reactive oxygen species (ROS), and ROS induce several DNA oxidative damages. Attributed to these characteristics, the observed mitochondrial dysfunction are categorized as either quantitative, including reduced mtDNA content or increased heteroplasmic mitochondria, or qualitative, such as different types of mtDNA mutations (7). In some animal models, the association between age-dependent decreased fertility and reduced mtDNA content in oocytes were reported in the older populations (15, 16). In humans, significantly lower mtDNA amounts were also found in women with advanced maternal age or diminished ovarian reserve (17).

Therefore, different approaches to detect and calculate mtDNA copies in the embryos were explored when trophectoderm biopsies were performed for PGT-A. Fragouli et al. first reported that the euploid embryos with lower mtDNA to nuclear DNA ratio (mtDNA ratio) tend to behave at a higher implantation rate in a total of 89 blastocyst transfers (2015) (18). Continuously, the same research team applied the criterion of “less is better” to mtDNA ratio in a total of 199 blastocyst transfers, and then illustrated that the ongoing pregnancy rate was lower in those with elevated mtDNA ratio (2017) (19). However, some articles did not support the mtDNA ratio as an independent parameter for implantation of embryo transfer, since the mtDNA ratios between implanted and non-implanted populations displayed more like a uniform pattern rather than a binarily classified pattern (20–22).

One of the challenges to reliability of mtDNA ratio as a selection tool in euploid embryo transfers is that the potential effects from other biological features remains unclear. Since the distribution of mitochondria from each oocyte to blastomeres of the subsequent embryo is random, the feasibility of applying mtDNA ratios across individual embryos with discrepant backgrounds must be carefully determined (23). The contribution derived from other biological features could mask the actual effect of mtDNA ratio. These features to mtDNA ratio must be comprehensively controlled before evaluating correlation between mtDNA ratio and transfer outcomes.

Furthermore, the utilized methodologies and calculation algorithms could also lead to different results (24, 25). The most common method to quantify mtDNA is qPCR assay, however, the additional experiment would be burdensome in the clinical PGT-A implementation. Thus different approaches were tested on the NGS platform that has been used in the PGT-A, such as increasing sequencing depth to obtain more reads (18) or employment of correction factors to normalize the backgrounds (20). Nevertheless, no consensus for the methodologies has been reached.

Increase of sequencing depth would raise the cost per sample, and employment of multiple correction factors must be validated in accordance with individual laboratories’ conditions. In the present study, we directly applied the bam files generated from the present PGT-A/NGS protocol to develop a reasonable algorithm for calculating mtDNA ratio with validations of reliability and repeatability, and then used the established mtDNA ratio in the analyses of biological effects. Eventually, the correlation between mtDNA ratios and the reproductive outcomes of blastocysts would be evaluated with controlled biological backgrounds.

## Materials and methods

### Study design

**Figure 1:**
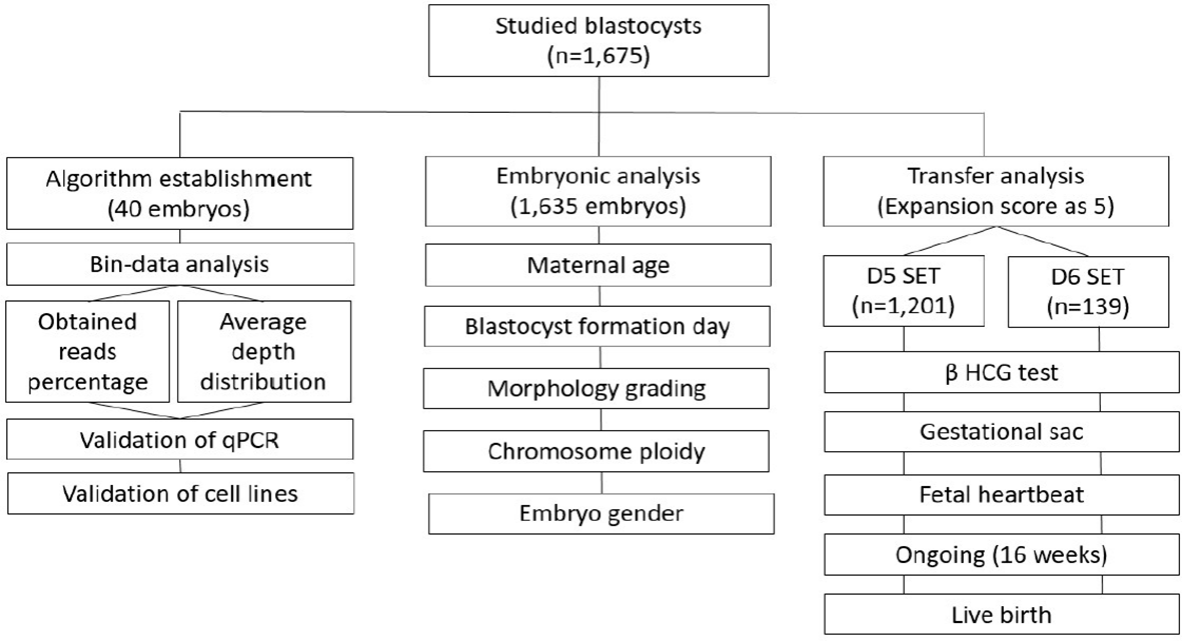
The workframe for study of mtDNA ratio is displayed. There are three stages: 1) establishment and validation of the algorithm for calculating mtDNA ratio; 2) estimation of the effect from biological features to mtDNA ratio; 3) evaluation of the correlation between reproductive outcomes and mtDNA ratios. SET, single embryo transfer; eSET, single euploid embryo transfer.

This retrospective study has been approved by an independent institutional review board of National Taiwan University Hospital. The entire workframe was displayed in **Figure 1**: first, to develop and validate a reasonable calculation method using a smaller cohort; second, to separately estimate the effect from different biological features to the established mtDNA ratios, including maternal age, day of blastocyst formation (day of biopsy), expansion stage, morphological grading, chromosome ploidy, and embryo gender, in a larger cohort; third, to evaluate the correlation between the reproductive outcomes and mtDNA ratios in the cycles with single euploid embryo transfer (eSET).

### Study subjects

A sequencing database generated from 1,675 blastocysts involving 1,305 individuals with IVF/PGT-A programs was analyzed for reproductive outcomes, collected between January 2015 to December 2019. A total of 1,389 blastocysts involving 1,201 individuals amongst the study cohort underwent eSET (**Supplementary Table 1**). All the involved patients underwent personalized stimulation protocols for the IVF treatment (26, 27). Written informed consents were obtained from the couples before entering the programs. The fertilized oocytes were cultured to the blastocyst stage, and then biopsied using mechanical shearing force between the biopsy and holding pipettes (Origio, Måløv, Denmark). Around 5-10 cells from trophectoderm (TE) were removed (28). Biopsy was only conducted in the blastocysts graded above BC according to the Gardner and Schoolcraft system (1999). The biopsied samples were washed twice in sterile 1X phosphate-buffered saline (PBS) solution (Cell Signaling Technologies, Danvers, MA, US) containing 1% (w/v) polyvinylpyrrolidone (PVP; Sigma, St. Louis, MO, US). Then the sample was gently transferred into a 0.2-mL PCR tube in 2.5 μL of PBS/PVP solution and stored at −20°C for following NGS procedures.

### Determination of chromosome ploidy *via* NGS

DNA extraction and amplification were applied with the Sureplex amplification system (Sureplex; Illumina, San Diego, CA, US). Initially samples were lysed with the extraction master mix. Then they were fragmented and primed using a pre-amplification cocktail. They were finally amplified using an amplification master mix. A Qubit dsDNA HS (high-sensitivity) Assay Kit (Qubit; Life Technologies, Waltham, MA, US) was used to quantify the concentration of amplified products. The products were diluted to 0.2 ng/μl for the library preparation according to the manufacturer’s guidelines of VeriSeq PGS v.3 (Illumina), and then sequenced using a Miseq System (Illumina). The generated data was analyzed *via* the BlueFuse Multi Software (Illumina). For the CNV calculation, the human reference sequence of individual chromosomes was divided into several intervals with unit lengths, so-called ‘bins’ in the Veriseq PGS system, and reads that pass quality metrics were mapped by the intervals. The chromosomal CNV were subsequently calculated using the variation of mapped reads across these bins. If a median chromosomal CNV deviated from the default CNV represented as two, a gain or loss of chromosome would be identified (29). Internal validations of sensitivity and specificity could be referred to our previous publications (30, 31). No default setting in the sequencing parameter was changed.

### Determination of mtDNA ratio *via* NGS

Bam files generated from the Veriseq PGS were utilized for calculating mtDNA ratios. Reads went through a self-developed QC metrics *via* the written commands on the pysam platform (Release 0.15.0) for filtering. Unmapped reads, duplicates, and reads with low quality scores or with poor performance of alignment were then excluded. Because normalizing the variations of biopsied cell numbers and of sequencing batches for relative mtDNA quantification were necessary, an adequate denominator derived from nucleic DNA was required. This denominator would be applied in the embryos with different backgrounds for the following analyses of biological features. Thus chromosome 6 was initially targeted due to its lowest incidence of aneuploidy across all somatic chromosomes (32). Since the ploidy identification was carried out according to the variation of bin count data, the denominator was applied with the same scale. The sequencing depth and distribution of alignments were then screened across individual bins on chromosome 6 in a total of randomly selected 40 embryos involving different sequencing runs. The particular bin(s) with the most reliable depths and stable percentage of obtained reads would be selected as the denominator. Finally, the reads of mtDNA would be normalized to that of selected bin(s) to calculate mtDNA ratio.

### Validation of mtDNA ratio *via* qPCR

To validate the reliability of mtDNA ratio established from the NGS platform, a second methodology using qPCR was performed. The former selected 40 embryos were tested by NovaQUANT Human Mitochondrial to Nuclear DNA Ratio Kit (Merck, Darmstadt, Germany). All the procedures were referred to the manufacturers’ user guide. Two pairs of mitochondrial gene and nuclear gene were calculated for the relative mtDNA ratios: ND1 and BECN1; ND6 and NEB. In the application, both the mitochondrial genes, ND1 and ND6, code the subunit of NADH dehydrogenase, which is the largest, highly conservative component of Complex I of mitochondrial respiratory chain; and the other two nuclear, single-copy genes, BECN1 and NEB code the highly conservative eukaryotic proteins (Beclin 1 and Nebulin), used as the factors for normalizing the biopsied cell number. Resultant *Cts* (Cycle threshold value, Ct) obtained from the qPCR assay can generate an average of *2^ΔCt^* from ND1 to BECN1, ND6 to NEB, *mean (2^ΔCt of ND1- BECN1^, 2^ΔCt of ND6- NEB^)*, for calculation of mtDNA copy number per cell (mtDNA ratio). Of manipulation, the WGA products were serially diluted to 0.1 ng/μL with DNase free water. Equal volume of 2X RT 2 Fast SYBR Green Mastermix (Thermo Fisher Scientific, Waltham, MA, USA) was mixed with a total of 1ng WGA products. Then 20μL of the mixed solution was transferred into a commercial qPCR plate separately coated with pre-aliquoted qPCR primers for individual ND1, ND6, BECN1, and NEB genes. Then qPCR assay was performed using a thermal cycler (QuantStudio 3 Real Time PCR System; Thermo Fisher Scientific), and the condition was as follows: incubation at 95°C for 1 min and then 40 cycles of 95°C for 15 sec and 64°C for 1 min. Relative mtDNA ratio was determined by an equation of 2^ΔCt^, and then the average between the two pairs of 2^ΔCt^ was calculated for the final measurement as above mentioned. Each sample was tested in triplicate.

### Validation of mtDNA ratio *via* cell lines

To validate the repeatability of mtDNA ratio among different batches on the NGS platform, three cell lines with different ploidies were applied. Two self-developed amniotic stem cell lines were kindly provided by the Bioresource Collection and Research Center (Hsinchu, Taiwan): AFMSC-T (46, XY), and AFMSC-9703125 (45, X0; Turner syndrome). One cell line was purchased from the Coriell Cell Repository (Camden, NJ, USA), AG12070 (47, XX, trisomy 13). The karyotypes of these cell lines were identified by the providers previously before utilization. The cell lines were passaged according to the suppliers’ guides, and then were diluted to 10-20 cells in PBS/PVP solution of 2.5 μL. A total of 10 replicates were prepared for each cell line. Samples of cell lines underwent the same procedures of amplification and library preparation as clinical biopsies. Finally, they were sequenced in 10 different NGS runs.

### Correlation between mtDNA ratios and biological features

The mtDNA ratios were compared by the following features of embryos: maternal age, day of blastocyst formation, expansion stage, grading of morphology (Gardner’s system), chromosome ploidy, and embryo gender. Individual comparison was performed for analyzing the possible effect from a particular feature, while the other features were controlled. Since the entire mtDNA ratios displayed a typical non-normal distribution (**Supplementary Figure 1**), a Kolmogorov-Smirnov test would be applied for confirming the distribution before conducting statistical comparisons.

### Correlation between mtDNA ratios and reproductive outcomes

All the included patients underwent hormone replacement therapy (HRT) for endometrial preparation, and transferred with a single euploid blastocyst. Average mtDNA ratios were analyzed according to the reproductive outcomes. Five endpoints during pregnancy were recorded: serum β-HCG test after two weeks of cryotransfer, detection of gestational sac (Sac) and fetal heartbeat (FHB) after four weeks of cryotransfer, ongoing pregnancy of 16 weeks, and full-term live birth (LB) after 36 weeks of pregnancy. Embryonic parameters of the analyzed cohorts would be controlled according to the conclusions obtained from the former analyses of biological features.

### Statistical methods

Before conducting any analyses, a Kolmogorov-Smirnov test was applied to confirm the normality. The Spearman’s rank correlation coefficient (Spearman’s ρ) was performed for evaluating correlation. For the continuous variables between two groups, Mann-Whitney test or Wilcoxon rank sum test were applied. For the continuous variables among multiple groups, Kruskal-Wallis test was used. All the statistics were accomplished using SPSS software (IBM; Armonk, NY, US).

## Results

### Establishment of mtDNA ratio

**Figure 2:**
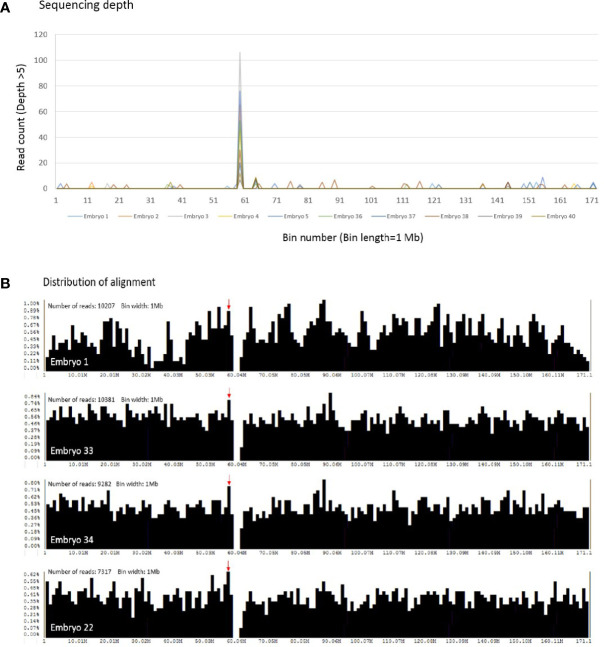
The sequencing depth and distribution of alignment across individual bin intervals on chromosome 6 are shown. **(A)** With the condition of sequencing depth above five, only a single bin contains exclusively higher read counts, and a peak is shown obviously in the randomly selected 40 embryos (10 embryos are represented in this figure). The x scale denotes the bin number on chromosome 6, and the y scale as the read counts of depth above five. **(B)** The distribution of alignment is screened in the selected embryos. A red arrow indicates the particular bin with higher read counts of depth above five, and upper left is the total read counts of chromosome 6 obtained in individual embryos. Each bar represents the percentage of obtained reads in a single bin interval. The x scale denotes the bin number on chromosome 6, and the y scale as the percentage of obtained reads.

Detailed information of randomly selected 40 embryos derived from 37 patients was displayed in **Supplementary Table 2**. Mean age of the involved patients was 36.5 years (range: 26-40 years). The studied cohort included 23 euploid embryos, 16 aneuploid embryos, and 1 mosaic embryo. The sequencing depth and distribution of alignments on chromosome 6 were analyzed across individual 1 Mb bin intervals in the testing embryos (**Figure 2**). **Figure 2A** elucidated the reads with sequencing depth above five, and only a single bin on chromosome 6 obtained exclusively higher read counts, shown as a significant peak in the line chart. Then the distribution of alignment was screened in **Figure 2B**. The particular bin with higher read counts of depth above five also exhibited a comparatively higher percentage of obtained alignments. Both the sequencing depth and distribution of alignments of this single bin were more stable than the other intervals. Therefore, the reads of mitochondrial DNA would be normalized by that of this particular bin on chromosome 6 for calculating mtDNA ratio.

### Validation of mtDNA ratio *via* qPCR

To evaluate the reliability of mtDNA ratio established from NGS methodology in the present study, a second methodology using qPCR assay was performed. Since the scales of the two methodologies are discrepant, correlation between the ratios derived from qPCR and NGS was illustrated in a log-log plot of **Supplementary Figure 2**. Mitochondrial DNA ratio derived from qPCR was calculated as *mean (2^ΔCt of ND1- BECN1^, 2^ΔCt of ND6- NEB^)* and that from NGS was $\frac{Read\;count\;\left(mtDNA\right)}{Read\;count\;\left(Chr.6\;target\;bin\right)}$ . Strong correlation was reached between two datasets of qPCR and NGS (Spearman ρ=0.706).

### Validation of mtDNA ratio *via* cell lines

To validate the repeatability of mtDNA ratio among different sequencing batches of the NGS platform, three cell lines with different ploidies were applied. Each cell line was tested in ten replicates within ten different sequencing runs, and the results were displayed as box plots (**Supplementary Figure 3**). Based on the calculation of precision-to-tolerance ratio (P/T), the established mtDNA ratios of ten replicates across different sequencing batches showed acceptable repeatability: [P/T of 46, XY]=15.7%; [P/T of 46,X0]=26.2%; [P/T of Trisomy 13]=17.0%.

### Correlation between mtDNA ratios and biological features

**Figure 3:**
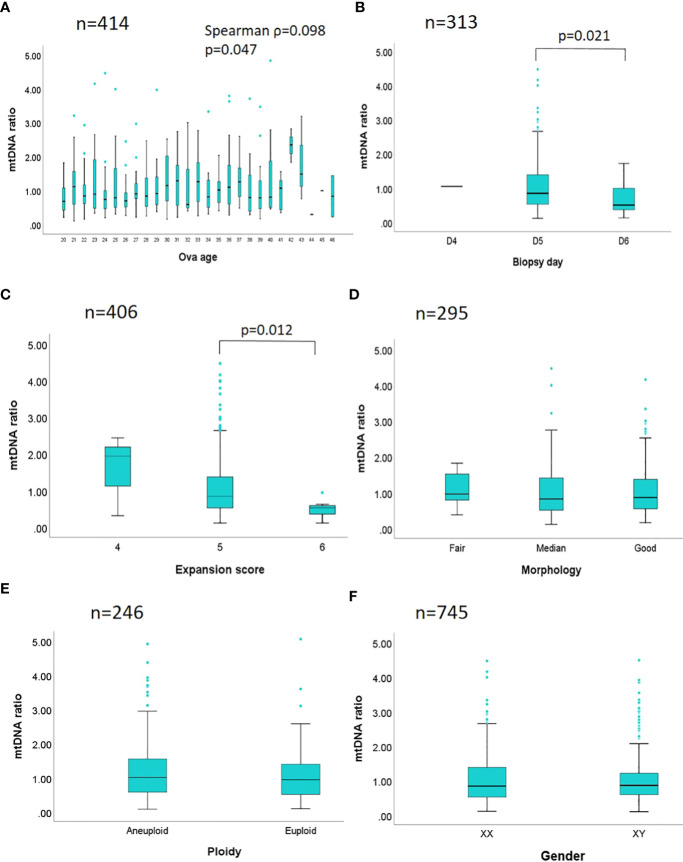
Stratification analyses between mtDNA ratio and biological features are performed. The mtDNA ratios of 1,635 embryos are compared by different biological features in six strata. **(A)** A total of 414 euploid embryos with a fixed day of blastocyst formation, expansion state, morphology grading, and gender, are analyzed by the maternal age. **(B)** A total of 313 euploid embryos with fixed age span, expansion state, morphology grading, and gender, are analyzed by the days of blastocyst formation. **(C)** A total of 406 euploid embryos with fixed age span, a fixed day of blastocyst formation, morphology grading, and gender, are analyzed by the expansion state. **(D)** A total of 295 euploid embryos with fixed age span, day of blastocyst formation, expansion state, and gender are analyzed by the morphology grading ([good]: AA, AB, BA; [median]: BB; [fair]: AC, BC; based on the Gardner’s system). **(E)** Pair-matched embryos derived from a total of 246 sibling oocytes with paired maternal age, day of blastocyst formation, expansion state, morphology grading, and gender, are analyzed by the chromosome ploidy. **(F)** A total of 745 euploid embryos with fixed age span, day of blastocyst formation, expansion state, and morphology grading are analyzed by the genders. Spearman correlation is applied in the analysis of maternal age and mtDNA ratio; while Mann-Whitney test (Wilcoxon rank sum test), and Kruskal-Wallis test are applied in the comparisons of two or above two datasets respectively. The p value is labeled while the statistical significance is reached.

In **Figure 3**, a stratification analysis of the mtDNA ratios derived from a total of 1,635 embryos was conducted according to the six biological features: maternal age, day of blastocyst formation, expansion stage, grading of morphology, chromosome ploidy, and embryo gender (**Supplementary Table 3**). To individually estimate the actual effect from a particular feature, the other features would be controlled across each stratum. In **Figure 3A**, the mtDNA ratios from a total of 414 euploid embryos (day 5 blastocyst, expansion score 5, graded above BB, female) were analyzed by the maternal age, and merely weak correlation was observed (Spearman ρ=0.098). In the analysis of day of blastocyst formation (**Figure 3B**), a total of 313 euploid embryos (maternal age<35 years, expansion score 5, graded above BB, female) showed significant difference in the mtDNA ratios between day 5 and day 6 blastocysts (p=0.021). In the analysis of expansion state (**Figure 3C**) of 406 euploid embryos (maternal age<38, graded above BB, female), significance also reached between the euploid embryos with expansion score 5 and 6 (p=0.012), and expansion state showed highly dependent on the days of blastocyst formation (embryos with expansion score 6 were all day 6 blastocysts, n=7). In the analysis of morphology grading of 295 euploid embryos (maternal age<35 years, day 5 blastocyst, expansion score 5, female) (**Figure 3D**), no significant differences in mtDNA ratios were observed among good, median, and fair embryos ([good]: AA, AB, BA; [median]: BB; [fair]: AC, BC; based on the Gardner’s system). Of the chromosome ploidy (**Figure 3E**), the mtDNA ratios between pair-matched euploid and aneuploid embryos derived from 246 sibling oocytes displayed no significant difference (paired maternal age, day of blastocyst formation, expansion score, morphology grading, and embryo gender). Then no difference was found in the mtDNA ratios between two different genders in 745 euploid embryos (maternal age<35 years, day 5 blastocyst, expansion score 5, graded above BB) (**Figure 3F**).

### Correlation between mtDNA ratios and reproductive outcomes

The profile of all the recruited eSETs was shown in **Supplementary Table 1**. A total of 1389 blastocysts were generated from 1201 individuals (mean age: 30.1 years), including 694 cycles using their own oocytes (49.96%) and 695 cycles using donated oocytes (50.04%). All the individuals underwent hormone replacement therapy (HRT) for endometrium preparation and euploid blastocyst cryotransfer. The overall biochemical pregnancy rate was 71.6%, implantation rate as 62.3%, clinical pregnancy rate as 57.6%, ongoing pregnancy rate as 54.6%, and live birth rate as 53.4%. Based on the former stratification analyses between mtDNA ratio and biological features, significant difference of mtDNA ratio was only observed in the embryos with different days of blastocyst formation and expansion scores. Additionally, the expansion score was highly dependent on the days of blastocyst formation, the embryos with expansion score 6 were all day-6 blastocysts while the other characteristics had been controlled. Thus the reproductive outcomes in a total of 1,340 cycles with single euploid embryo cryotransfers were divided by the days of blastocyst formation with fixed expansion score as 5, and then the reproductive outcomes of each endpoint were analyzed separately. In the stratum of day 5 blastocysts (expansion score 5), **Table 1** displayed the mtDNA ratios by the outcomes of β-HCG, Sac, FHB, ongoing pregnancy (16 weeks), and LB in a total of 1,201 cycles with day 5 eSET. In the stratum of day 6 blastocysts, the same endpoints were evaluated in a total of 139 cycles with day 6 eSET with expansion score 5 (**Table 2**). Of the cycles with day 5 eSET, the maternal age, endometrium thickness, distribution of morphology, and mtDNA ratios between all the successful and failure groups showed no significant differences. Of the day 6 eSET, mtDNA ratios showed significant differences between the successful and failure groups in the detection of fetal heartbeat (p=0.004), ongoing pregnancy at 16 weeks (p=0.007), and live birth (p=0.01), while the other parameters were statistically similar. Additionally, a forward stratification to analyze the clinical outcomes by the quartiles of mtDNA ratios also displayed poor outcomes of fetal heartbeat, ongoing pregnancy, and live birth associated with lower mtDNA quantiles in the cohort of day 6 eSET (**Supplementary Tables 4**, **5**). Therefore, in the day 6 eSET, reduced mtDNA ratios were observed in the failure group, and no significant correlation was found in the day 5 eSET.

**Table 1:** Reproductive outcomes of cryotransfer with day 5 single euploid embryo (expansion score 5).

Transfer outcome	Success rate	Cycle number (n=1,201)	Maternal age (years)	EM thickness(mm)	Morphology grading (Good, Median, Fair)	mtDNA ratio (Mean, SD)	mtDNA ratio(Median, IQR)	P-value* for mtDNA ratio
HCG (+)	73.77%	886	30.07	9.57	286, 540, 60 (32%, 61%, 7%)	1.06 ± 0.77	0.86, 0.75	0.26
HCG (–)		315	29.87	9.33	89, 211, 15 (28%, 67%, 5%)	1.01 ± 0.69	0.86, 0.76	
Sac (+)	64.11%	770	29.97	9.65	248, 472, 50 (32%, 61%, 6%)	1.06 ± 0.77	0.86, 0.75	0.42
Sac (–)		431	30.10	9.27	127, 279, 25 (29%, 65%, 6%)	1.03 ± 0.70	0.86, 0.76	
FHB (+)	59.70%	717	29.94	9.65	231, 438, 48 (32%, 61%, 7%)	1.07 ± 0.79	0.86, 0.75	0.29
FHB (–)		484	30.14	9.30	144, 313, 27 (30%, 65%, 6%)	1.02 ± 0.69	0.85, 0.74	
Ongoing (+)	54.62%	656	29.93	9.66	210, 400, 46 (32%, 61%, 7%)	1.08 ± 0.80	0.87, 0.75	0.11
Ongoing (–)		545	30.12	9.32	165, 351, 29 (30%, 64%, 5%)	1.01 ± 0.68	0.84, 0.76	
LB (+)	53.12%	638	29.96	9.67	202, 390, 46 (32%, 61%, 7%)	1.08 ± 0.80	0.87, 0.74	0.18
LB (–)		563	30.09	9.33	173, 361, 29 (31%, 64%, 5%)	1.02 ± 0.68	0.84, 0.78	

EM, endometrium; Sac, gestational sac; FHB, fetal heartbeat; Ongoing, pregnancy at 16 weeks; LB, live birth; IQR, interquartile range. (+), success at the pregnancy endpoint; (-), failure at the pregnancy endpoint.

*P-value is calculated from Mann-Whitney U test for the mtDNA ratios between successful and failure groups.

**Table 2:** Reproductive outcomes of cryotransfer with day 6 single euploid embryo (expansion score 5).

Transfer outcome	Success rate	Cycle number (n=139)	Maternal age (years)	EM thickness(mm)	Morphology grading (Good, Median, Fair)	mtDNA ratio (Mean, SD)	mtDNA ratio(Median, IQR)	P-value* for mtDNA ratio
HCG (+)	60.43%	84	29.49	9.62	17, 52, 15 (20%, 62%, 18%)	0.68 ± 0.44	0.56, 0.58	0.77
HCG (-)		55	31.87	9.58	9, 39, 7 (16%, 71%, 13%)	0.66 ± 0.42	0.62, 0.49	
Sac (+)	53.24%	74	29.01	9.79	14, 47, 13 (19%, 64%, 18%)	0.71 ± 0.45	0.62, 0.62	0.18
Sac (-)		65	32.05	9.40	12, 44, 9 (18%, 68%, 14%)	0.62 ± 0.40	0.48, 0.38	
FHB (+)	46.04%	64	28.69	9.69	12, 41, 11 (19%, 64%, 17%)	0.78 ± 0.45	0.72, 0.57	0.004
FHB (-)		75	31.92	9.54	14, 50, 11 (19%, 67%, 15%)	0.58 ± 0.39	0.46, 0.31	
Ongoing (+)	38.85%	54	28.31	9.78	10, 35, 9 (19%, 65%, 17%)	0.79 ± 0.48	0.72, 0.62	0.007
Ongoing (-)		85	31.78	9.49	16, 56, 13 (19%, 66%, 15%)	0.59 ± 0.38	0.47, 0.40	
LB (+)	38.13%	53	28.36	9.74	10, 34, 9 (19%, 64%, 17%)	0.78 ± 0.48	0.71, 0.61	0.01
LB (-)		86	31.71	9.52	16, 57, 13 (19%, 66%, 15%)	0.60 ± 0.38	0.47, 0.41	

EM, endometrium; Sac, gestational sac; FHB, fetal heartbeat; Ongoing, pregnancy at 16 weeks; LB, live birth; IQR, interquartile range. (+), success at the pregnancy endpoint; (-), failure at the pregnancy endpoint.

*P-value is calculated from Mann-Whitney U test for the mtDNA ratios between successful and failure groups.

## Discussion

The study first demonstrated that mtDNA ratio was merely dependent on the day of blastocyst formation and the size of embryo. Reduced success rates were observed in the day 6 single euploid embryo cryotransfers involving reduced mtDNA ratio. Initially, the calculation algorithm was constructed from the bam files generated *via* present PGT-A/NGS procedures, validating by qPCR and cell lines assays. Then the established mtDNA ratio was stratified by different biological features for comparisons in blastocysts. The mtDNA ratios showed statistically different between embryos with different days of blastocyst formation, and the expansion stage. None or weakly correlated with the other features. Eventually, the mtDNA ratios with different days of blastocyst formation and fixed expansion stage (expansion score 5) in the cycles of eSET were analyzed by the different endpoints of pregnancy. Correlation between reduced mtDNA ratio and reduced success rates were observed in the day 6 euploid blastocysts. However, the result was opposite to the conclusion derived from the previous articles, which stated the adverse correlation between increased mtDNA ratio and reduced success rates (6, 18, 19).

Since the PGT-A technology has been used in the IVF programs, the feasibility of mtDNA ratio derived from the same biopsied sample was studied for further embryo selection. Diez-Juan et al. (2015) found that mtDNA ratios displayed differences between implanted and non-implanted populations of day 3 and day 5 embryos *via* qPCR assay (6). At the same time, Fragouli et al. (2015 and 2017) investigated mtDNA ratios between different maternal age, chromosome ploidy, and transfer outcomes *via* both qPCR and NGS assays (18, 19). They both reported that a better reproductive outcome was observed in the embryos with lower mtDNA ratios, and explained the phenomenon as an outcome of compensation to energetic stress in blastocysts. On the contrary, Victor et al. (20) analyzed mtDNA ratio by qPCR and NGS assays again according to the same variables with Fragouli et al. (19) in a comparatively large dataset, suggesting that mtDNA ratio reveals uniform between implanted and non-implanted populations (20). In the same year, Treff et al. (2017) reported that mtDNA ratios do not distinguish between sibling embryos that were implanted and not implanted through qPCR assay (22). More recently, a study involving a larger cohort conducted by Scott et al. (2020) also demonstrated that the mitochondrial DNA content is not a reliably predictive biomarker for reproductive outcomes in paired-sibling euploid blastocysts *via* qPCR measurement (33). No matter which methodologies were used to establish mtDNA ratio, the discrepant conclusions implied that the role of mitochondria quantity in the early-stage embryos may not be a simply independent parameter, and thus made its application in the embryo selection controversial.

Unlike the analyses of biological effect to mtDNA ratio in the previous articles compared the cohorts with mixed embryonic backgrounds, especially the mixed days of blastocyst formation and expansion stage, we investigated the biological effect to mtDNA ratio through stratification analyses in a larger cohort with a size of 1,637 embryos, estimating the actual interaction between an individual feature and mtDNA quantity in the present study. Except the embryos with different days of blastocyst formation and expansion stage displayed a significant difference in mtDNA ratios, no significance was reached across individual stratum regarding the other features. Because the timeline of embryo development seemed crucial to both the embryonic size and mitochondria quantity, the subsequent analyses of reproductive outcomes and mtDNA ratios were thus separated by the days of blastocyst formation with a fixed expansion score. The final results displayed a positive correlation between mtDNA ratio and pregnancy success rates in the population of day 6 single euploid blastocyst cryotransfers while the other parameters were controlled, suggesting that the embryonic timeline and mtDNA quantity are covariate.

Mitochondrial dysfunction could be divided as qualitative and quantitative, and mtDNA ratio was one of a quantitative measurement for mitochondrial copy number. In the studies of animal models, the correlation between reproductive aging and mitochondrial copy number decline were observed in the mice and cow models (15, 16). Additionally, introductions of mutants in mitochondrial transcriptional factor A (TFAM), which regulates mitochondrial copy number, displayed significant impact on animal embryo viability (34, 35). In human embryo studies, lower mtDNA content was also found in the oocytes of women with diminished ovarian reserve (36), and it is associated with compromised fertilization and development issues (17). However, a recent article (2022) elucidated that neither the mtDNA content nor the specific mtDNA genetic variants in cumulus cells was correlated with following ART outcomes *via* analyzing a huge sequencing dataset, implying that possible tolerance existed between the oocytes and their supportive environment (37). Due to manifestation of correlation between decreased mtDNA dosage and poorer developmental competence, increasing the quantity of functional mitochondria such as mitochondrial replacement therapies (MRT) exhibited as a potential treatment to the patients with inherited or infertility problems (7, 38). Since a sufficient amount of mitochondria has been reported as essential to oocyte fertilization and zygote development, giving priority to the embryos with lower mtDNA content in blastocyst selection seemed contrary to the former knowledge, and so far no enough evidence for the benefit was proved.

According to the analysis of mtDNA ratio and transfer outcomes in the present study, mtDNA ratio may have some but not strong connection with the pregnancy success rate in euploid embryos. While the embryonic sizes were controlled, the mtDNA ratio reduced with the success rate in day 6 eSET; and it displayed no significant correlation in the reproductive outcomes of day 5 eSET. The correlation among reduced mtDNA ratio, extended developmental timeline, and larger embryonic size were also observed in the several articles (39, 40), associating the previously observed phenomenon of continuously diluting the existing mtDNA copy number during the post-fertilization stage due to ceasing of mtDNA replication (14, 35). In the eSETs of present study, the first quantile range of mtDNA ratio in day 5 blastocysts was close to the second quantile range in day 6 blastocysts. The median in each quantile range of mtDNA ratios remains approximately 1.68 times higher in day 5 to day 6 populations (**Supplementary Figure 4**), possibly reflecting the total cell number difference of trophectoderm between the two populations (41). On the other hand, if the expansion stage had been furtherly fixed, namely, controlling the cell number in the same day of blastocyst formation, mtDNA ratio merely displayed the correlation between the embryonic timeline and initial mtDNA content of the particular oocytes. Therefore, it could play a similar role as the time-dependent parameters of development, which have been validated as applicable biomarkers for predicting the pregnancy outcomes (42). More information could be obtained by linking the mtDNA ratio and detailed morphokinetic data *via* time-lapse culture system in the future investigations.

To date, no consensus in the calculation of mtDNA ratio has been reached. Both additional qPCR assay or increasing NGS depth to obtain more mitochondrial reads required intervention in the original PGT-A/NGS implementation. In this study, a calculation algorithm was established from a commercial Veriseq PGS procedure, and then it was validated through qPCR and cell lines for the reliability and repeatability. Applied with this algorithm, two extremely high values were obtained in a total of 1,637 studied embryos, and they were filtered out in the analyses due to the possible events of allelic drop-out (ADO). Although the established algorithm showed stable performance in the analyses of this study, bias from ADOs could not be excluded. To screen the bins with sequencing depth above five across all somatic chromosomes, and combine them as a normalization factor, is applicable for future modifications.

Nevertheless, the study is limited to its retrospective nature. Evaluation of the relative mtDNA quantity lacks the evidence of gene integrity and functional assessment. Mitochondria display uniparental inheritance to reduce heteroplasmy, exhibiting a genetic bottleneck to prevent possible deleterious mutants in offspring (43, 44). However, mtDNA has a higher mutation frequency due to the insufficient repairing system and higher oxidative stress, the effect derived from these heteroplasmic mitochondria involving *de novo* variants in early-stage embryos derived from women without inherited mitochondrial disease remained uncertain. Deep sequencing or other functional assays in blastocyst biopsies may be useful to determine the role of mitochondria in human embryos more extensively. On the practical side, application of machine learning-based technologies to analyze the weighting of mtDNA quantity among multivariate databases of embryo selection could provide another data-driven approach before clinical implementation.

## Conclusions

In conclusion, mtDNA ratio is highly dependent on the days of blastocyst formation while the expansion stage was fixed, and it could reflect the immediate cell division progress. Reduced mtDNA ratio associated with reduced reproductive outcomes in the day 6 single euploid blastocyst transfers, but showed no significant correlation in the day 5 single euploid blastocyst transfers. Analysis of mtDNA quantity and developmental timeline in early-stage embryos is worthy for further investigation.

## Data availability statement

The original contributions presented in the study are included in the article/**Supplementary Material**. Further inquiries can be directed to the corresponding author.

## Ethics statement

The studies involving human participants were reviewed and approved by National Taiwan University Hospital, Research Ethics Committee. The patients/participants provided their written informed consent to participate in this study.

## Author contributions

T-HC designed the study and wrote the manuscript. C-YeC conducted the experiments and statistical works. C-SK built the bioinformatics pipelines. H-HL, C-LH, M-JL, and Y-TL, recruited the patients. Y-JC prepared the cell lines. C-YuC reviewed the bioinformatics and statistical results. S-UC revised the final manuscript, and approved the submitted version. All authors contributed to the article and approved the submitted version.

## Supplementary material

Supplementary Figure 1 The diagram displays the distribution of mtDNA ratios calculated from 1,673 embryos, and two outliers are excluded due to out of scale. A Kolmogorov-Smirnov test is applied for confirming normality. The skewness and kurtosis are 4.95 and 51.61, respectively (p<0.05).

Supplementary Figure 2 Since the scales between qPCR and NGS are very different, correlation between mtDNA ratios derived from two methodologies is displayed in a log-log plot. The x scale denotes the log values of mtDNA ratios derived from the NGS platform, and the y scale as that derived from the qPCR platform.

Supplementary Figure 3 Three cell lines with different ploidies are tested on the NGS platform for evaluating the repeatability of established mtDNA ratio. Ten replicates of each cell line involve ten different sequencing runs.

Supplementary Table 1 Profile of patients for correlation analysis.

Supplementary Table 2 Profile of embryos for algorithm establishment.

Supplementary Table 3 Profile of embryos for correlation analysis.

Supplementary Table 4 Reproductive outcomes of cryotransfer with day 5 single euploid embryo (expansion score 5).

Supplementary Table 5 Reproductive outcomes of cryotransfer with day 6 single euploid embryo (expansion score 5).
