# Supplementary material for: Reduced mitochondrial DNA content correlate with poor clinical outcomes in cryotransfers with day 6 single euploid embryos
Source: Front Endocrinol (Lausanne). 2023 Jan 4;13:1066530. doi: 10.3389/fendo.2022.1066530 (PMC9846089; doi:10.3389/fendo.2022.1066530)
Supplement: Supplementary Figure 1 — The diagram displays the distribution of mtDNA ratios calculated from 1,673 embryos, and two outliers are excluded due to out of scale. A Kolmogorov-Smirnov test is applied for confirming normality. The skewness and kurtosis are 4.95 and 51.61, respectively (p<0.05). [file Image_1.pdf]

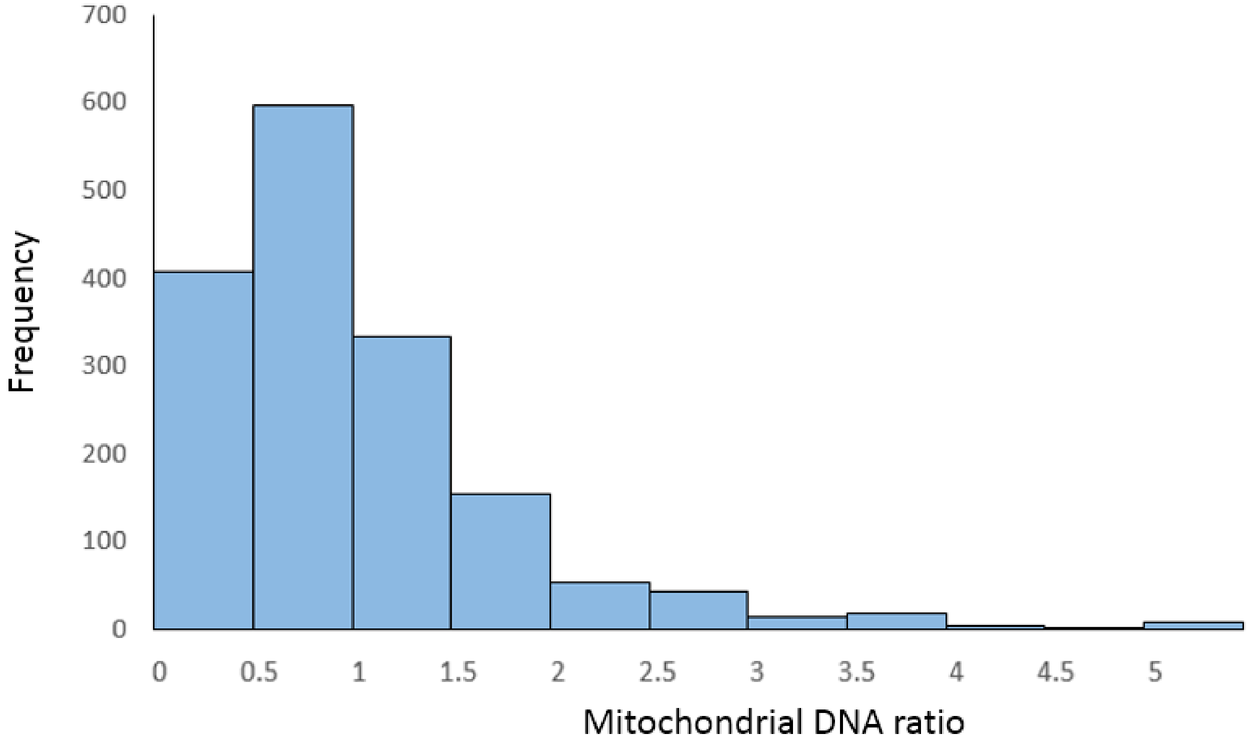

Supplementary Figure 1

The diagram displays the distribution of mtDNA ratios calculated from 1,673 embryos, and two outliers are excluded due to out of scale. A Kolmogorov-Smirnov test is applied for confirming normality. The skewness and kurtosis are 4.95 and 51.61, respectively ($p < 0.05$).
