# Supplementary material for: Reduced mitochondrial DNA content correlate with poor clinical outcomes in cryotransfers with day 6 single euploid embryos
Source: Front Endocrinol (Lausanne). 2023 Jan 4;13:1066530. doi: 10.3389/fendo.2022.1066530 (PMC9846089; doi:10.3389/fendo.2022.1066530)
Supplement: Supplementary Figure 2 — Since the scales between qPCR and NGS are very different, correlation between mtDNA ratios derived from two methodologies is displayed in a log-log plot. The x scale denotes the log values of mtDNA ratios derived from the NGS platform, and the y scale as that derived from the qPCR platform. [file Image_2.pdf]

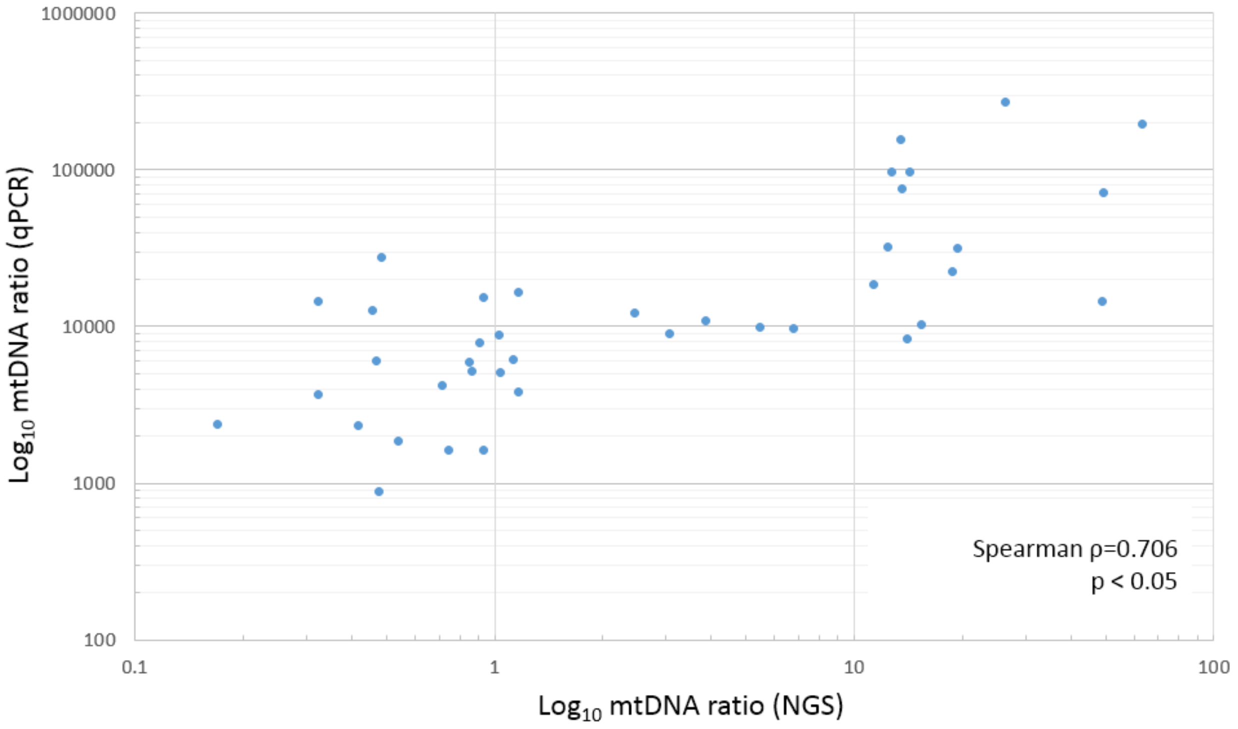

Supplementary Figure 2

Since the scales between qPCR and NGS are very different, correlation between mtDNA ratios derived from two methodologies is displayed in a log-log plot. The x scale denotes the log values of mtDNA ratios derived from the NGS platform, and the y scale as that derived from the qPCR platform.
