# Supplementary material for: Reduced mitochondrial DNA content correlate with poor clinical outcomes in cryotransfers with day 6 single euploid embryos
Source: Front Endocrinol (Lausanne). 2023 Jan 4;13:1066530. doi: 10.3389/fendo.2022.1066530 (PMC9846089; doi:10.3389/fendo.2022.1066530)
Supplement: Supplementary Figure 3 — Three cell lines with different ploidies are tested on the NGS platform for evaluating the repeatability of established mtDNA ratio. Ten replicates of each cell line involve ten different sequencing runs. [file Image_3.pdf]

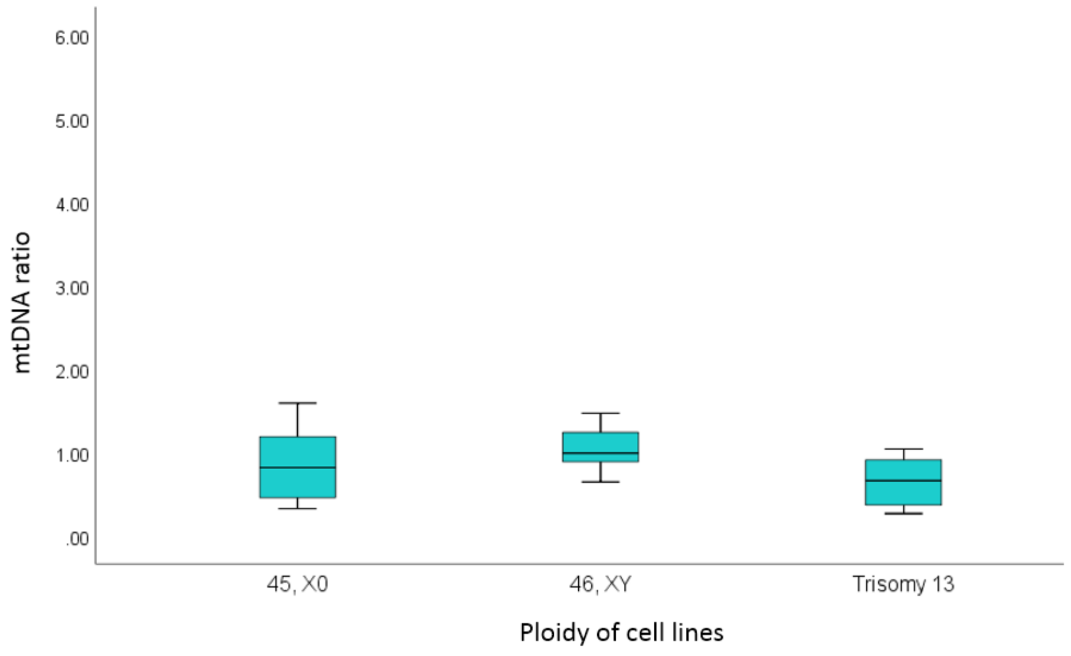

Supplementary Figure 3

Three cell lines with different ploidies are tested on the NGS platform for evaluating the repeatability of established mtDNA ratio. Ten replicates of each cell line involve ten different sequencing runs.
