# Supplementary material for: Reduced mitochondrial DNA content correlate with poor clinical outcomes in cryotransfers with day 6 single euploid embryos
Source: Front Endocrinol (Lausanne). 2023 Jan 4;13:1066530. doi: 10.3389/fendo.2022.1066530 (PMC9846089; doi:10.3389/fendo.2022.1066530)
Supplement: Supplementary file 4 [file Image_4.pdf]

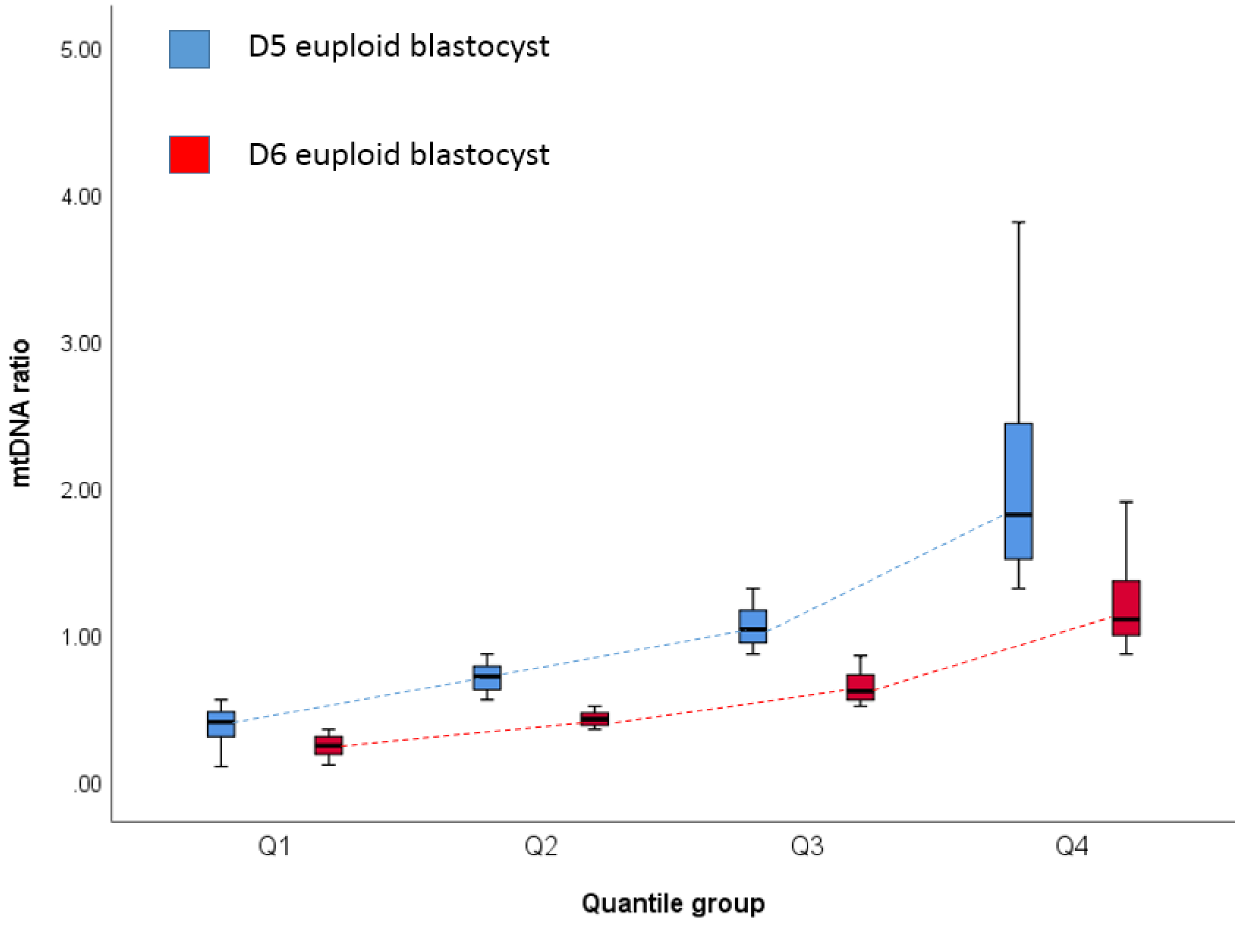

Supplementary Figure 4

The distribution of mtDNA ratio across quantile ranges between day 5 and day 6 euploid blastocysts are displayed in the box plots. The differences of median between day 5 and day 6 blastocysts are 1.67 times (Q1), 1.69 times (Q2), 1.69 times (Q3), and 1.65 times (Q4), respectively.
