# Supplementary material for: Reduced mitochondrial DNA content correlate with poor clinical outcomes in cryotransfers with day 6 single euploid embryos
Source: Front Endocrinol (Lausanne). 2023 Jan 4;13:1066530. doi: 10.3389/fendo.2022.1066530 (PMC9846089; doi:10.3389/fendo.2022.1066530)
Supplement: Supplementary Table 1 — Profile of patients for correlation analysis. [file Table_1.pdf]

Supplementary Table 1 Profile of patients for correlation analysis

Patient number	1201
Mean age, years (SD)	30.10 (6.80)
Age span (years)	20-46
eSET[a] cycle	1389
Oocyte source	
Using own oocytes, n(%)	694 (49.96%)
Using donated oocytes, n(%)	695 (50.04%)
Indication of PGT-A	
Advanced maternal age (≥ 36 years)	317 (22.82%)
Repeated pregnancy failure	377 (27.14%)
Intention to eSET	695 (50.04%)
EM[b] preparation	HRT[g]
EM thickness (nm)	9.53
Initial serum progesterone (ng/ml)	0.33
HCG(+) cycle number	995
Sac[c] (+) cycle number	865
FHB[d](+) cycle number	800
16 wk[e] (+) cycle number	758
LB[f] cycle number	741
Biochemical pregnancy rate	71.63%
Implantation rate	62.28%
Clinical pregnancy rate	57.60%
Ongoing pregnancy rate	54.57%
Live birth rate	53.35%

[a]Single euploid embryo transfer

[b]Endometrium

[c]Gestational sac

[d]Fetal heartbeat

[e]Ongoing pregnancy to 16 week

[f]Live birth

[g]Hormone replacement therapy
