# Supplementary material for: Reduced mitochondrial DNA content correlate with poor clinical outcomes in cryotransfers with day 6 single euploid embryos
Source: Front Endocrinol (Lausanne). 2023 Jan 4;13:1066530. doi: 10.3389/fendo.2022.1066530 (PMC9846089; doi:10.3389/fendo.2022.1066530)
Supplement: Supplementary Table 2 — Profile of embryos for algorithm establishment. [file Table_2.pdf]

Supplementary Table 2 Profile of embryos for algorithm establishment

Patient number	37
Mean age (SD, years)	36.50 (4.14)
Mean age, years (SD)	26-44
Embryo number	40
Blastocyst formation day	
Day 5	34 (85%)
Day 6	6 (15%)
Expansion	
5	40 (100%)
Morphology	
AA, AB, BA	3 (7.5%)
BB	27 (67.5%)
BC	10 (25%)
Ploidy	
Euploid	23 (57.5%)
Mosaicism*	1 (2.5%)
Aneuploid	16 (40%)
Gender	
Male	24 (60%)
Female	16 (40%)

*Mosaicism, 20-80% aneuploidy
