# Supplementary material for: Reduced mitochondrial DNA content correlate with poor clinical outcomes in cryotransfers with day 6 single euploid embryos
Source: Front Endocrinol (Lausanne). 2023 Jan 4;13:1066530. doi: 10.3389/fendo.2022.1066530 (PMC9846089; doi:10.3389/fendo.2022.1066530)
Supplement: Supplementary Table 3 — Profile of embryos for correlation analysis. [file Table_3.pdf]

Supplementary Table 3 Profile of embryos for correlation analysis

Patient number	1276
Mean age (SD, years)	30.26 (6.73)
Mean age, years (SD)	20-46
Embryo number	1635
Blastocyst formation day	
Day 4	1 (0.06%)
Day 5	1471 (89.97%)
Day 6	163 (9.97%)
Expansion score	
4	16 (0.98%)
5	1591 (97.31%)
6	28 (1.71%)
Morphology	
AA, AB, BA	468 (28.62%)
BB	1034 (63.24%)
BC	133 (8.13%)
Ploidy	
Euploid (eSET*)	1390
Paired euploid-aneuploid (sibling oocytes)	246 (123 pairs)
Gender	
Male	616 (37.68%)
Female	1019 (62.32%)

*Single euploid embryo transfer
