# Supplementary material for: Reduced mitochondrial DNA content correlate with poor clinical outcomes in cryotransfers with day 6 single euploid embryos
Source: Front Endocrinol (Lausanne). 2023 Jan 4;13:1066530. doi: 10.3389/fendo.2022.1066530 (PMC9846089; doi:10.3389/fendo.2022.1066530)
Supplement: Supplementary Table 4 — Reproductive outcomes of cryotransfer with day 5 single euploid embryo (expansion score 5). [file Table_4.pdf]

Supplementary Table 4 Reproductive outcomes of cryotransfer with day 5 single euploid embryo (expansion score 5)

mtDNA ratio	Q1 (0.1-0.55)	Q2 (0.55-0.86)	Q3 (0.86-1.31)	Q4 (1.31-6.42)	P-value
Cycle number	300	300	300	30	-
Mean age (SD, years)	30.4 (7.0)	29.4 (6.5)	29.8 (6.8)	30.5 (6.5)	0.14
EM thickness (mm)	9.4	9.5	9.5	9.7	0.25
Morphology, n (%)					
Good	87 (29.0)	96 (32.0)	96 (32.0)	96 (31.9)	0.87
Median	196 (65.3)	184 (61.3)	182 (60.7)	189 (62.8)	
Fair	17 (5.7)	20 (6.7)	22 (7.3)	16 (5.3)	
β HCG(+)	211	232	214	229	0.14
β HCG(-)	86	68	86	72	
β HCG(+) rate	70.3%	77.3%	71.3%	76.1%	
Sac(+)	183	202	187	198	0.34
Sac(-)	117	98	113	103	
Sac(+) rate	60.7%	66.8=9%	63.0%	65.6%	
FHB(+)	171	185	175	186	0.54
FHB(-)	129	115	125	115	
FHB(+) rate	57.0%	61.7%	58.3%	61.8%	
16wk(+)	157	177	169	170	0.24
16wk(-)	143	123	131	121	
Ongoing rate	52.3%	59.0%	56.3%	59.8%	

LB(+)	154	176	165	172	0.30
LB(-)	146	124	135	129	
Live birth rate	51.3%	58.7%	55.0%	57.1%	

EM, endometrium; Sac, gestational sac; FHB, fetal heartbeat; 16wk, 16 weeks of pregnancy; LB, live birth
