# Supplementary material for: Reduced mitochondrial DNA content correlate with poor clinical outcomes in cryotransfers with day 6 single euploid embryos
Source: Front Endocrinol (Lausanne). 2023 Jan 4;13:1066530. doi: 10.3389/fendo.2022.1066530 (PMC9846089; doi:10.3389/fendo.2022.1066530)
Supplement: Supplementary Table 5 — Reproductive outcomes of cryotransfer with day 6 single euploid embryo (expansion score 5). [file Table_5.pdf]

Supplementary Table 5 Reproductive outcomes of cryotransfer with day 6 single euploid embryo (expansion score 5)

mtDNA ratio	Q1 (0.12-0.36)	Q2 (0.36-0.52)	Q3 (0.53-0.91)	Q4 (0.93-2.05)	P-value
Cycle number	35	35	35	34	-
Mean age (SD, years)	29.5 (7.0)	31.6 (6.3)	31.1 (7.7)	29.5 (6.7)	0.45
EM thickness (mm)	9.5	9.6	10.2	9.2	0.19
Morphology, n (%)					
Good	3 (8.6)	7 (20.0)	6 (17.1)	10 (29.4)	0.31
Median	24 (68.6)	24 (68.6)	25 (71.4)	18 (52.9)	
Fair	8 (22.9)	4 (11.4)	4 (11.4)	6 (17.6)	
b-HCG(+)	24	17	22	21	0.37
b-HCG(-)	11	18	13	13	
b-HCG(+) rate	68.6%	48.6%	62.9%	61.8%	
Sac(+)	19	14	20	21	0.30
Sac(-)	16	21	15	13	
Sac(+) rate	54.3%	40.0%	57.1%	61.8%	
FHB(+)	15	8	20	21	0.005
FHB(-)	20	27	15	13	
FHB(+) rate	42.9%	22.9%	57.1%	61.8%	
16wk(+)	13	7	17	20	0.008
16wk(-)	22	28	18	14	

Ongoing rate	37.1%	20.0%	48.6%	58.8%	
LB(+)	13	7	17	19	0.014
LB(-)	22	28	18	15	
Live birth rate	37.1%	20.0%	48.6%	55.9%	

EM, endometrium; Sac, gestational sac; FHB, fetal heartbeat; 16wk, 16 weeks of pregnancy; LB, live birth
